# Comparison of frozen and RNALater solid tissue storage methods for use in RNA expression microarrays

**DOI:** 10.1186/1471-2164-5-88

**Published:** 2004-11-10

**Authors:** George L Mutter, David Zahrieh, Chunmei Liu, Donna Neuberg, David Finkelstein, Heather E Baker, Janet A Warrington

**Affiliations:** 1 Department of Pathology, Brigham and Women's Hospital, Boston, MA, USA; 2 Department of Biostatistical Science, Dana Farber Cancer Institute, Boston, MA, USA; 3 Affymetrix Inc, Santa Clara, CA, USA

## Abstract

**Background:**

Primary human tissues are an invaluable widely used tool for discovery of gene expression patterns which characterize disease states. Tissue processing methods remain unstandardized, leading to unanswered concerns of how to best store collected tissues and maintain reproducibility between laboratories. We subdivided uterine myometrial tissue specimens and stored split aliquots using the most common tissue processing methods (fresh, frozen, RNALater) before comparing quantitative RNA expression profiles on the Affymetrix U133 human expression array. Split samples and inclusion of duplicates within each processing group allowed us to undertake a formal genome-wide analysis comparing the magnitude of result variation contributed by sample source (different patients), processing protocol (fresh vs. frozen vs. 24 or 72 hours RNALater), and random background (duplicates). The dataset was randomly permuted to define a baseline pattern of ANOVA test statistic values against which the observed results could be interpreted.

**Results:**

14,639 of 22,283 genes were expressed in at least one sample. Patient subjects provided the greatest sources of variation in the mixed model ANOVA, with replicates and processing method the least. The magnitude of variation conferred by processing method (24 hours RNALater vs 72 hours RNALater vs. fresh vs frozen) was similar to the variability seen within replicates. Subset analysis of the test statistic according to gene functional class showed that the frequency of "outlier" ANOVA results within each functional class is overall no greater than expected by chance.

**Conclusions:**

Ambient storage of tissues for 24 or 72 hours in RNALater did not contribute any systematic shift in quantitative RNA expression results relative to the alternatives of fresh or frozen tissue. This nontoxic preservative enables decentralized tissue collection for expression array analysis without a requirement for specialized equipment.

## Background

Many of the hopes for achieving clinical benefits of genomic medicine will hinge on the ability to develop an efficient specimen conduit from clinic to laboratory. Quantitative gene expression studies have created unprecedented tissue collection and handling challenges. In particular, the rapid degeneration of RNA, and possible perturbation of expression following excision place a high premium on prompt stabilization of tissue samples intended for expression analysis. This can be accomplished by sending a dedicated trained technologist outfitted with the necessary specialized equipment, such as liquid nitrogen, into the clinical environment. Alternatively, clinicians can be enabled to process the specimens directly in the course of patient care and send them in some stable form by unrushed and routine means for centralized processing. The latter is greatly preferred when patients are physically dispersed, and becomes essential in a multi-institutional setting.

High throughput quantification of RNA expression in solid tissues has become a commonplace modality for genome-wide discovery of mechanisms of disease. Typically, groups of samples classified into comparison groups are used as a training set for expression pattern discovery, followed by validation in a fresh challenge set of annotated cases. The likelihood of success is highly dependent on the accuracy of classification within the training set, and ability to control random variables introduced during tissue processing and analytical measurement of RNA abundance. Efforts to standardize RNA quantification include sharing of information regarding probe design and use [1], or centralized design and production of analytical reagents and platforms by commercial entities using good manufacturing procedures (GMP).

Flash freezing, either by immersion in liquid nitrogen or on dry ice, is the most common means of stabilizing tissue samples intended for RNA analysis. Local access to the necessary materials and expense of cold shipping and/or storage limit these collection capabilities in most clinical settings. An additional disadvantage of frozen storage is that homogenization of frozen tissue must be accomplished rapidly to avoid the rapid RNA degeneration that occurs during thawing of a previously frozen sample.

Room temperature immersion of fresh tissue samples in aqueous sulfate salt solutions (such as ammonium sulfate) at controlled pH precipitates degenerative RNAses [2] and other solubilized proteins, thereby preserving the tissue with intact RNA [3]. Tissues preserved in this manner are compatible with most RNA isolation protocols, and may be archivally stored for extended periods at -60°C. A commercial preparation of this preservative, RNALater (Ambion), is increasingly being used by individual investigators and cooperative groups [4] for collection of human tissues. There have been promising reports of microarray-based RNA expression studies using RNALater-preserved tissues [5-10]. Solid tissues stored for a week in RNALater at room temperature give comparable RNA yields, and specific gene RNA abundance as with frozen tissue[8]. RNA yields are not affected substantially by storage at room temperature compared to 4°C, for storage intervals up to 3 months [11]. RNALater preserved tissues and cell suspensions are suitable starting points for RNA quantification by quantitative RT-PCR [11] and expression microarray hybridization [12]. One shortcoming of the prior work is that the potential changes contributed by RNALater use have not been precisely measured relative to random processing effects.

**Figure 1:**
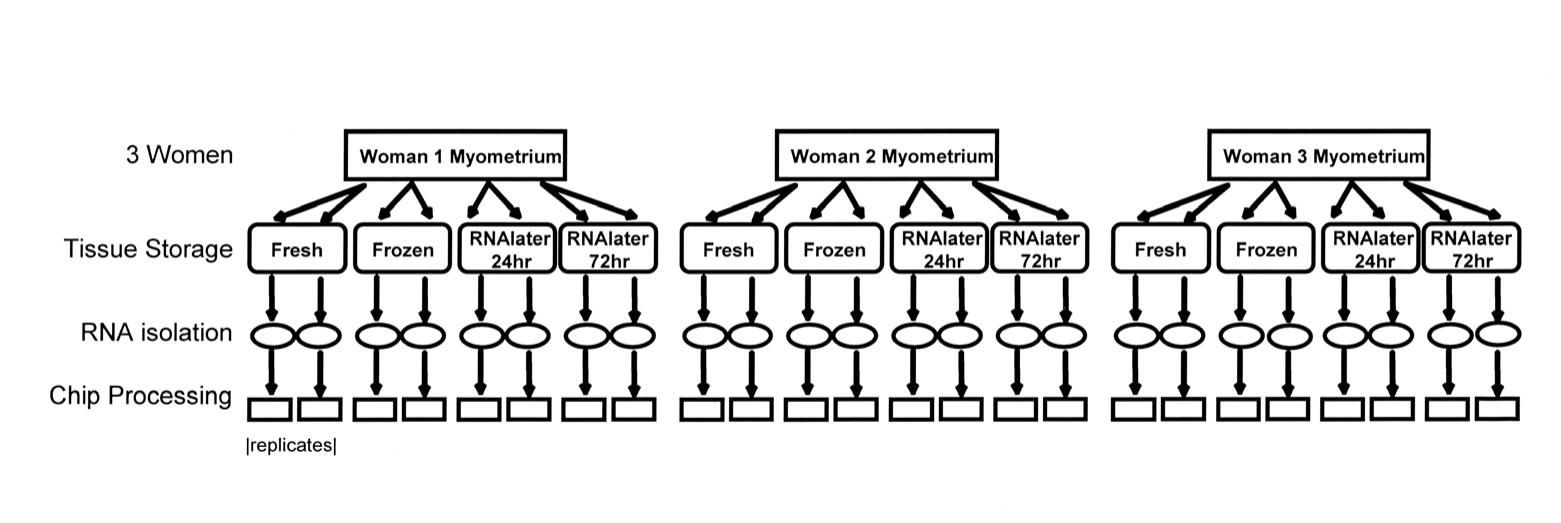
**Experimental design. **Tissue aliquots from 3 women were aliquoted, in duplicate, into four storage groups before RNA isolation and microarray hybridization. ANOVA design elements including fixed (storage group), random (woman, duplicate processing), and random interactive (woman × storage) effects as listed in Table 1.

We studied the effects of differences between storage conditions on gene expression as measured by expression array. Duplicate uterine myometrial tissue samples from three women were processed under each of 4 fixed storage conditions – fresh, frozen, 24 hours RNA-later and 72 hours RNA-later. The 24 labeled cRNA samples (Figure 1) were hybridized to HG-U133A Affymetrix microarrays. Then, for each microarray a data matrix was generated of 22,283 probe sets (genes) by quantitative expression levels in each RNA sample, and the effect of subject source, tissue processing, and replicates (Table 1) determined by ANOVA. Subset analysis by gene functional class was then performed to determine if storage condition has a specific effect on particular groups of genes.

**Table 1:** Variability Sources in ANOVA Model (See Figure 1). Mixed ANOVA Model:Xij = u + ai + Bj + Eij where Xij is the observation (LN intensity), ai is the tissue storage effect, Bj is the individual variability effect and Eij is the noise term.

**Variability Source**	**Type**	**df**
Tissue Storage	Fixed	3
Woman, Individual Variation	Random	2
Interaction (Woman × Storage)	Random	6
Replication (RNA isolation, chip Processing)	Random	12

We found no systematic bias in measured quantitative level of gene expression by processing method, indicating that short term storage in RNALater is a valid alternative to traditional frozen storage.

## Results

Of the 22,283 genes, 14,639 did not have absolutely null expression across all 24 samples. We fit the mixed model ANOVA from their log values and recorded this F statistic. The permutation distribution was used to assess the significance of F statistics calculated for each gene in the dataset. In this approach all 13,824 or (4!)^3 ^possible ways of permuting 4 pairs of replicate samples within each subject were considered. For each of these, the F statistics were computed for each gene. To control the overall error rate, the distributions of the maximum F statistics over the genes were used. That is, for each gene, the p-value is the proportion of permutations with the maximum F statistics over all genes greater or equal to the observed value for a particular gene. A test declaring as significant any genes with p < 0.05 then guarantees that the chance of any false positives being selected is < 5%. Similar analyses were performed replacing the distribution of the maximum F statistic with the distribution of the F statistics at the 95^th ^percentile and then at the 90^th ^percentile. After closer examination of the 387 genes in the 5% tail, we noted that most were exhibiting expression values below 100 for all 24 samples. In fact, within a storage condition, 2 out of 3 patients exhibited null expression while the third patient showed expression values other than null but less than 100 for at least one of their replicates. Therefore, as an additional analysis, any expression values less than 100 were recoded as 100. Genes that showed expression levels of 100 across all 24 samples, and therefore lacked variability, were then removed from the analysis. This resulted in 7,853 genes for which there was at least one sample with expression level greater than 100 across all 24 samples.

**Figure 2:**
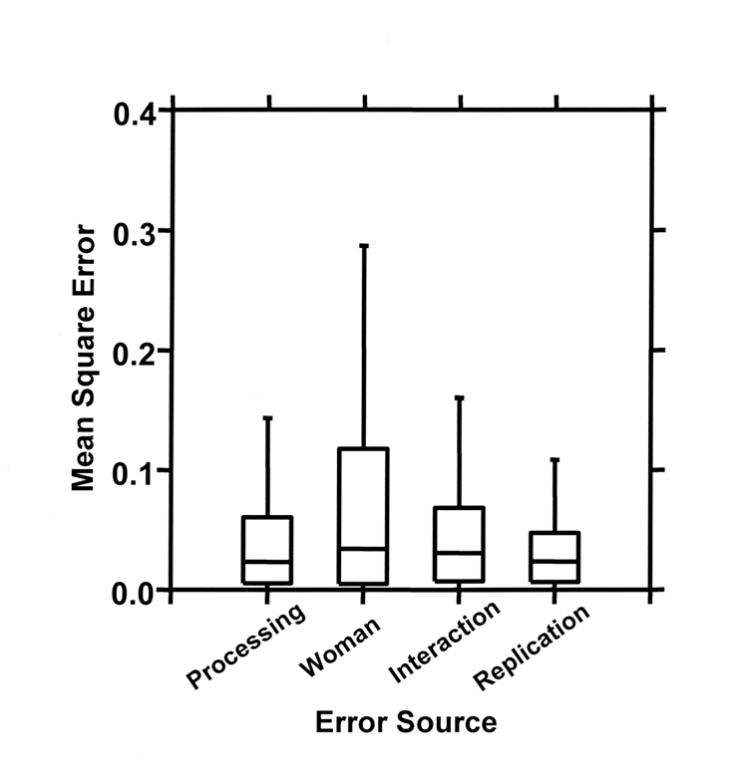
**Sources of variation in the mixed ANOVA model**. Distribution of Mean Squares Errors by variation source are plotted for those genes with at least one tissue showing expression at a level above LN(100) (7853 genes). Note that individual women emerge as the dominant source of variation. Variation contributed by tissue storage is of approximately the same magnitude as that seen between duplicate samples within the same storage group. Boxes encompass inner quartiles, horizontal line represents the median or the second quartile, and whiskers delimit 1.5 times the interquartile range. Because of the very large number of data points, outliers were suppressed in this summary plot.

Patient subjects provided the greatest sources of variation in the mixed model ANOVA, with replicates and processing method the least (Figure 2). The magnitude of variation conferred by processing method (24 hours RNALater vs 72 hours RNALater vs. fresh vs frozen) was similar to the variability seen within replicates. This is strong evidence that those individual expression profiles characteristic of the source tissue (woman) are unlikely to be obscured by the small amount of variation introduced by the processing method chosen.

**Figure 3:**
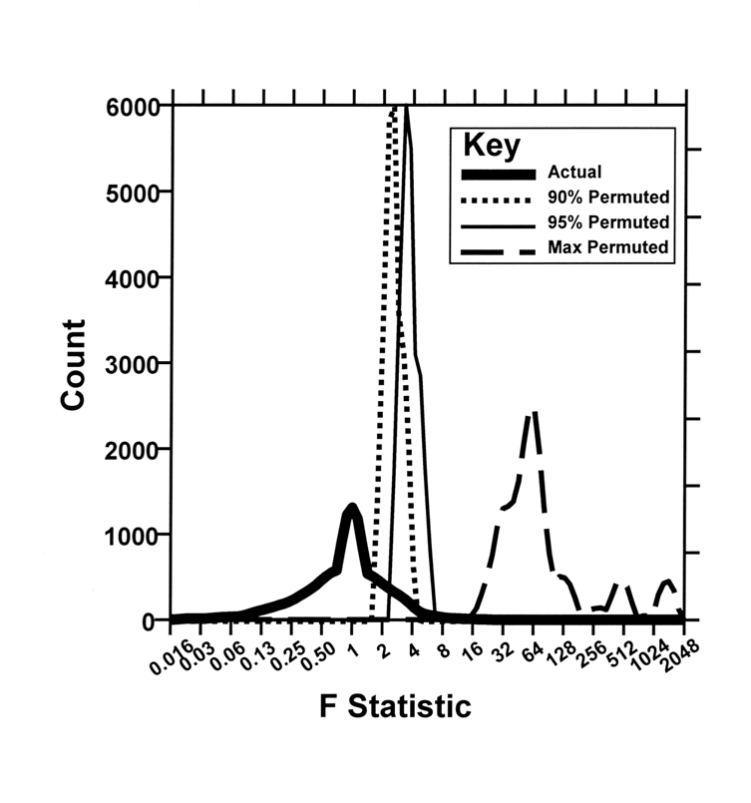
**Distribution of actual test statistics vs. randomly permuted background. **Distribution of ANOVA F-statistics from the model shown in Figure 1 were calculated for the observed (FStat Observed) and permuted datasets. The maximum, 95th percentile, and 90th percentile F-Statistics in the permuted dataset provide an index of the distribution of test results expected for a random sample. The values of test statistics seen at the 95% level in a randomly permuted dataset are greater than those of the observed dataset. Genes were included if at least one tissue showed expression above LN(100) (7853 genes).

The distribution of ANOVA test statistics on those 7,853 genes where at least one sample of 24 had expression at a level exceeding 100 is compared in Figure 3 to those seen in the randomly permuted dataset. In the actual dataset, the maximum observed F statistic was 25.52; the observed F statistic at the 95th percentile was 3.51; and the observed F statistic at the 90^th ^percentile was 2.58. Corresponding p-values were 0.94, 0.55 and 0.51, respectively. The values of test statistics seen at the 95% level in a randomly permuted dataset (Figure 3, thin solid line) are greater than those of the observed dataset (Figure 3, thick solid line). This indicates that the model variation contributed by processing method is of the same magnitude as that seen randomly.

**Figure 4:**
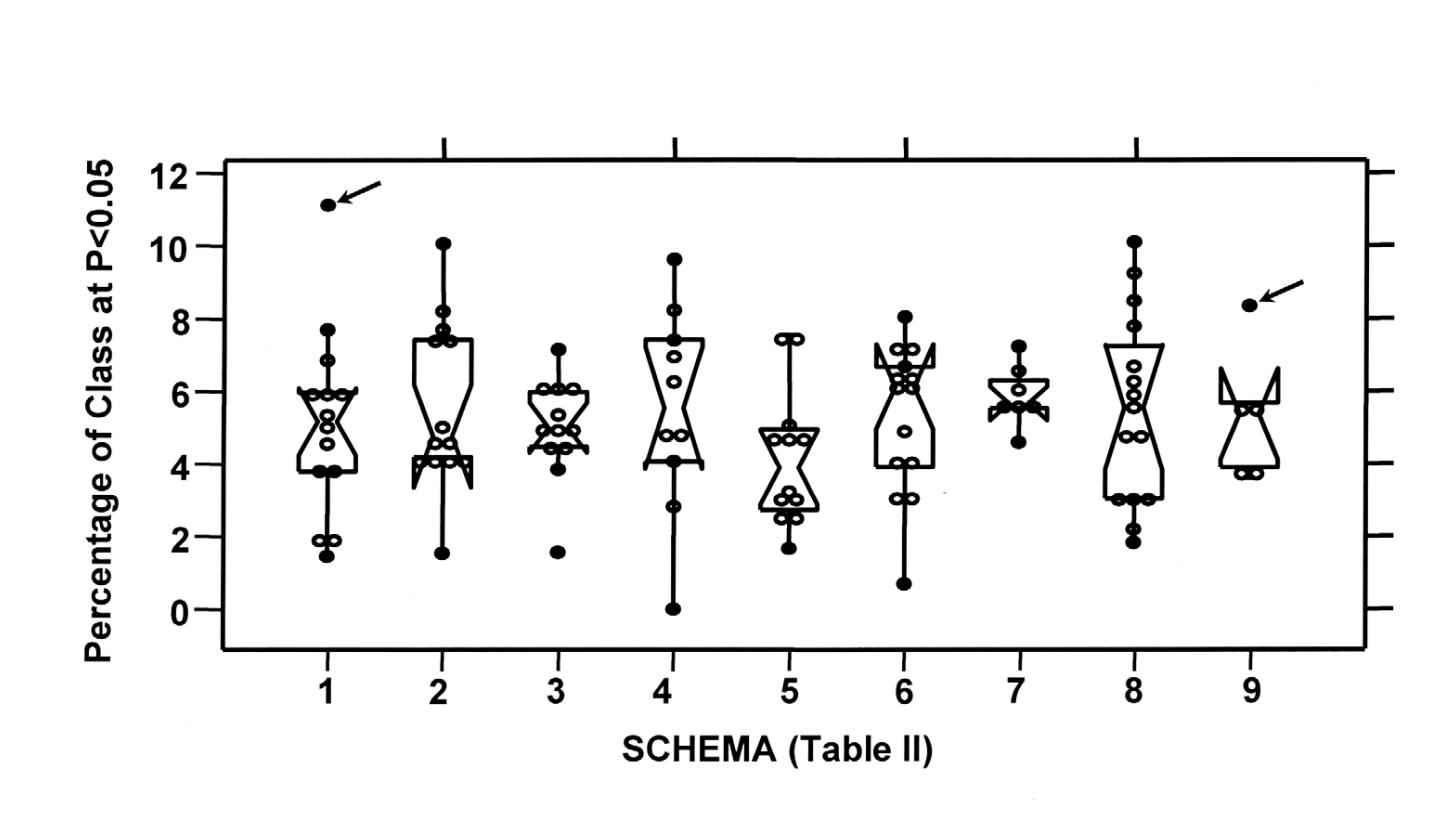
**Proportion of functional gene classes in which the test statistic falls within the nominal p < 0.05 tail. **Nine publically available gene functional annotation schemas (listed in Table 2), each composed of multiple functional gene classes, were used to determine if specific functional subsets of expressed genes were more likely to show significant change in RNA detection between tissue treatments. Percentage of individual gene classes with test statistics above the nominal p = 0.05 level (F Statistic >3.51 in the dataset of 7853 expressed genes) are plotted on the Y axis for each of 9 classification schema (X axis). Results for functional classes of genes with a minimum of 50 available expressed genes are shown as individual data points (circles) the distribution of which is summarized for each schema by the superimposed notch plot. There are only two high outliers (arrows) amongst the 113 gene classes shown. These are a messenger RNA splicing factors in the Biological Process (GO) schema (Schema 1), and translation factor in the Pathways (GenMAPP) schema (Schema 9). This frequency of 2/113 outliers is no greater than expected by chance, (>5).

Subset analysis of the test statistic according to gene functional class (Table 2) showed that the frequency of "outlier" ANOVA results within each functional class is overall no greater than expected by chance. Test statistic distribution was compiled by functional annotation for those 7853 genes which had at least one sample with detectable expression above a level of 100. Nine separate classification schemas containing a total of 127 functional classes were studied. Probe sets were rank ordered by decreasing ANOVA test statistic, and enrichment in the nominal p = 0.05 tail (F>3.51, containing top 387 of 7853 expressed) plotted by functional class (circles) and schema (box plot) (Figure 4).

**Table 2:** Test statistic results by functional class within 9 annotation schema. Amongst 9 schema, a total of 127 functional classes ("Classes") contained expressed genes ("Total Genes"), and of these, 102 classes had a sufficient number of expressed genes (>50, "Class>50") to enumerate ("Genes F>3.51") and calculate fractional representation ("%>3.51", also Figure 3) in the nominal p = 0.05 tail at a test statistic cutoff of 3.51. 7853 genes with expression in at least one tissue >LN(100) were included.

Code	Schema	Classes	Class>50	TotalGenes	Genes F>3.51	%>3.51
1	Biological Process (GO)	22	14	3591	197	5.5
2	Cellular Role (Proteome)	14	13	1775	92	5.2
3	Cellular Component (GO)	15	12	2845	154	5.4
4	Molecular Localization (Proteome)	9	10	1788	93	5.2
5	Organismal Role (Proteome)	17	12	1524	64	4.2
6	Biochemical Function (Proteome)	16	14	2727	150	5.5
7	Subcellular localization (Proteome)	8	7	2047	116	5.7
8	Molecular Function (GO)	21	15	4473	239	5.3
9	Pathways (GenMAPP)	6	5	756	33	4.4

## Discussion

Storage of fresh whole human tissues for up to 72 hours at room temperature in RNALater does not introduce quantitative bias into RNA expression determinations with the Affymetrix U133A array. Several differing standards justify this conclusion. First, by construction of a test model (Figure 1) incorporating both random reproducibility estimates (replicate determinations) and between-sample differences it has been possible to demonstrate that the magnitude of variation introduced by RNALater processing is equivalent to that seen within routine repeat specimens in a common processing group (Figure 2). Second, the extent of result variation conferred by RNALater processing is not statistically significant when measured against the randomly permuted dataset (Figure 3). This is an important element in evaluation of large datasets in which small numbers of individual variables may randomly demonstrate extreme values of the test statistic. Lastly, there is no evidence that specific functional subgroups of genes have aberrant behavior in this regard (Figure 4).

Uterine myometrium was selected for these experiments because its components (myocytes, fibroblasts, vascular elements) are evenly intermingled throughout the myometrial compartment, lending itself to physical subdivision into equivalent aliquots. This would not be possible with more complex tissues in which differing cell types are distributed asymmetrically within the specimen. Despite the equivalency of subdivided fractions that underwent varying storage treatments, it must be noted that this is a hormonally responsive tissue whose expression patterns would be expected to differ between individual women as a function of monthly changes in circulating sex hormones. We did not control for hormonal factors or indication for hysterectomy (prolapse or fibroids) but selected patients randomly. It comes as no surprise that expression differences between women, irrespective of processing method, emerged as the dominant source of inter-sample variation. This was anticipated in constructing the model, by assigning the subject source of specimens as a random variable which could be measured against the fixed processing effects. It is likely that if a larger number of women had been included in the study, the observed biologic variation attributable to subject would have been even greater. Since our goal was to compare magnitude of variation contributed by subjects to that conferred by processing method, we achieved a balanced design by having comparable degrees of freedom for those two variables.

There are several critical procedural elements that must be highlighted for successful preservation of solid tissues in aqueous sulfate salt solutions such as RNALater. These reagents enter the tissue through passive diffusion, a process which follows simple physical principles. The distance between the tissue surface, which is exposed to preservative, and the innermost regions of the fragment should be minimized. We did this by cutting the tissues into 2 mm thick slices, thereby reducing the diffusion distance to 1 mm or less. Clumping of multiple fragments into a mass that excludes preservative may obviate the benefits of fine division. This can be avoided either by gentle agitation or placement in a sufficiently large container that individual pieces are likely to disperse. Results reported here are for tissues stored at room temperature (23–25°C). Storage under cooler conditions (4°C) as recommend by the manufacturer of RNALater were not directly evaluated in this experiment because it was our intent to mimic storage interval and conditions commonly encountered when sending a specimen by express courier to a centralized processing facility. Storage at temperatures substantially higher than 25°C, especially before the preservative has had an opportunity to penetrate the tissue, should be avoided.

## Conclusions

Split samples of fresh human tissue yield quantitatively similar RNA expression profiles whether processed fresh, frozen, or following 24–72 hour storage in RNALater. Formal statistical analysis shows patient source is the predominant source of variation between samples, with processing method contributing a random level of variation comparable to that seen in split duplicates (replicates). Subset analysis by functional gene category did not identify a specific class of genes which responded differently by processing method.

Use of nontoxic ambient environment tissue preservatives makes it practical to engage practicing clinicians directly in decentralized sample collection for high throughput expression analysis in a central location. Tissue handling closely resembles that used by clinicians to prepare specimens for routine pathology analysis. Upon receipt in a centralized facility, the samples can either be immediately homogenized or archived at -60°C.

## Methods

### Tissue handling and storage

Normal fresh uterine myometrial tissues were collected randomly from three women undergoing hysterectomy for benign uterine disease. For each hysterectomy, a single 4 to 8 gram tissue fragment was subdivided into eight aliquots composed of thin slices measuring no more than 2 mm in thickness. Replicate aliquots were immediately triaged into one of four storage conditions prior to homogenization: 1)immediate homogenization; 2)flash frozen in liquid nitrogen and storage for 48 hours at -80°C; 3)24 hour immersion in RNALater at room temperature with gentle agitation; or 4)72 hours immersion in RNALater at room temperature with gentle agitation.

### RNA isolation

Tissue was solubilized in Trizol reagent (Gibco BRL, Grand Island, NY), and RNA isolated according to the manufacturers instructions. In brief, the aqueous phase was resolved by addition of chloroform, and RNA precipitated from the aqueous phase by addition of isopropyl alcohol. Pelleted RNA was washed with 70% ethanol, dried, and resuspended in water. Quality of total RNA was assessed by running a non-denaturing 1% agarose tris-acetate buffer which confirmed the integrity of 18S and 28S ribosomal bands for all 24 total RNA preparations.

### Microarray chip hybridization and data normalization

Double-stranded cDNA was generated from 8 μg total RNA using the Superscript Choice System (Life Technologies) with T7-(dT)24 oligomer. cDNA was purified by phenol/chloroform extraction and ethanol precipitation. Biotin-labeled cRNA was prepared using the Enzo BioArray HighYield RNA Transcript labeling kit (Affymetrix). Unincorporated NTPs were removed from the biotinylated cRNA using an RNeasy kit (Qiagen). 10 μg of quality, fragmented cRNA was hybridized to each Affymetrix HG-U133A arrays containing probe sets representing approximately 22,000 genes. Array hybridization, washing was done according to the manufacturer's protocol (Affymetrix, GeneChip^® ^Expression Analysis Technical Manual) and all arrays were scanned under a low PMT (Photo Multiplier Tube) of 570 nm. Global scaling to a target value of 75 was applied to normalize all the arrays so they were comparable (Affymetrix Microarray Analysis Suite MAS5.0). The Affymetrix average-difference expression data and the P/A calls were used in the analysis. Those probe sets determined to have no detectible signal above background mismatch hybridization (Call of "Absent") were assigned a nominal value of 1 to facilitate future log transformations. Probesets having at least one tissue with detectable expression (call of "present") and an average difference above either 1 or 100 were selected to define subsets of 14639 permissively or 7853 stringently expressed genes, respectively. Further analysis was performed using the natural log transformed data of these probe subsets. Data files for all specimens processed are deposited online at the Gene Expression Omnibus at the National Center for Biotechnology Information [13].

### Biostatistical analysis

For this two factor study, a mixed model analysis of variance (ANOVA)was used, regarding storage condition as a fixed factor with four levels and subject as a random factor with three levels. The analysis of variance calculations for sums of squares in the mixed model ANOVA are identical to those for the fixed ANOVA model. Similarly, the degrees of freedom and mean squares are exactly the same. The mixed ANOVA model departs from the fixed ANOVA model only in the expected mean squares and the consequence choice of the appropriate test statistic. The mixed model also included a random storage by subject interaction. Replicate samples enabled us to estimate the replication error in the model. To test for the presence of storage main effects for each gene we divided the mean square for storage by the mean square for the interaction effect between storage and subject [14]. The ANOVA test statistic was calculated using C++.

Functional annotation of probesets on the U133A chip, were downloaded from the Netaffx^tm ^download center [1]. The March, 2003 version matches individual probesets with functional annotations (Table 2) from public domain databases including: the Gene Microarray Pathway Profiler, Gene Ontology Consortium, Proteome BioKnowledge Library, and Kyoto Encyclopedia of Genes and Chromosomes. Within each schema (comprised of many functional classes of genes), each gene is assigned to a primary functional class. Each probe set may be represented in several different schemas. Individual functional classes with at least 50 probesets represented within the U133A array were plotted by schema to show fractional representation within the nominal 0.05 tail (Figure 4). This provides a rapid and intuitive manner to identify functional classes of genes biased towards high test statistics in the ANOVA model.

## Authors' contributions

GM and JW conceived and designed the research plan and participated in all aspects of data collection and analysis. DF participated in data analysis and interpretation. DN and DZ performed the statistical analysis. CL and HB performed the RNA isolations, chip hybridizations, and data collation.
